# Correction: The synthesis of calcium arsenate@iron arsenate coating materials and their application for arsenic-containing wastewater treatment

**DOI:** 10.1039/d0ra90005b

**Published:** 2020-01-22

**Authors:** Yang Wang, Zhihao Rong, Xincun Tang, Shan Cao

**Affiliations:** College of Chemistry and Chemical Engineering, Central South University Changsha 410083 China tangxincun@csu.edu.cn; School of Light Industry and Engineering, Qilu University of Technology Shandong 250353 China cs1988@qlu.edu.cn

## Abstract

Correction for ‘The synthesis of calcium arsenate@iron arsenate coating materials and their application for arsenic-containing wastewater treatment’ by Yang Wang *et al.*, *RSC Adv.*, 2020, **10**, 719–723.

The authors regret that there were errors in the Characterization of samples section of the original article, in Section 3.1 on page 720 of the original article. These errors are detailed below.

The sentence beginning, “As is known, the main peaks of scorodite (FeAsO_4_·2H_2_O) are located at approximately 30.1°, 27.8°, 16.9° and 10.9°…” should read, “As is known, the main peaks of CaHAsO_4_ are located at approximately 30.1°, 27.8°, 16.9° and 10.9°, which can be indexed as the (221), (220), (101) and (020) lattice planes of CaHAsO_4_ (JCPDS no. 18-0288), respectively.”

The sentence beginning, “As is known, the main peaks of scorodite located at approximately 30.8° and 31.7°…” should read, “As is known, the main peaks of Ca_3_(AsO_4_)_2_ located at approximately 30.8° and 31.7°, can be indexed as the (020) and (021) lattice planes of Ca_3_(AsO_4_)_2_ (JCPDS no. 01-0933).”

The Royal Society of Chemistry apologises for these errors and any consequent inconvenience to authors and readers.

## Supplementary Material

